# Video consultations in oculoplastic service: a continuing role post-pandemic?

**DOI:** 10.1038/s41433-023-02385-3

**Published:** 2023-03-01

**Authors:** Ourania Fydanaki, Tavishi Kanwar, Minak Bhalla, Mana Rahimzadeh, Hussein Ibrahim, Rajni Jain, Ahmad Aziz, Vickie Lee

**Affiliations:** 1 Western Eye Hospital, Imperial College Healthcare Trust, 153-173 Marylebone Rd, London, NW1 5QH UK; 2 Guy’s and St Thomas’ NHS Foundation Trust, Westminster Bridge Road, London, SE1 7EH UK

**Keywords:** Health services, Signs and symptoms

Video consultations (VCs) increased across specialties during the Covid-19 pandemic, as they allow social distancing. In the US, Ophthalmology had the greatest decline in outpatient visits early in the pandemic [1]. Oculoplastic VCs have proved successful, partly due to emphasis on visual recognition [2]. A comparison of face-to-face consultations (F2FC) with photographs assessed independently showed 100% detection rate for extraocular malignancy [3]. Previous studies on VC uptake were done during a period when concerns of catching Covid-19 in hospitals ran high, possibly influencing patient and clinician responses [2]. As social distancing relaxes, we describe how VCs are being used in Oculoplastics in 2022.

We trialed VCs in the Oculoplastic service at Imperial Healthcare NHS Trust. The primary aims were to evaluate the efficacy; patient and clinician satisfaction and preference to telephone consultation (TCs) or F2FC. We included consultations for patients attending as new referrals, follow-up and pre/post-operative.

The VCs were conducted using the DrDoctor (ICNH Ltd) platform. The interface can be accessed on web browsers on smartphones, tablets or computers. For clinicians, there is two-factor authentication. Clinicians had access to patient standard electronic patient records simultaneously. After the VC, we conducted a questionnaire on patient and clinician experience and use of technology. We collated answers using Microsoft Excel and analysed with descriptive statistics.

**Fig. 1:**
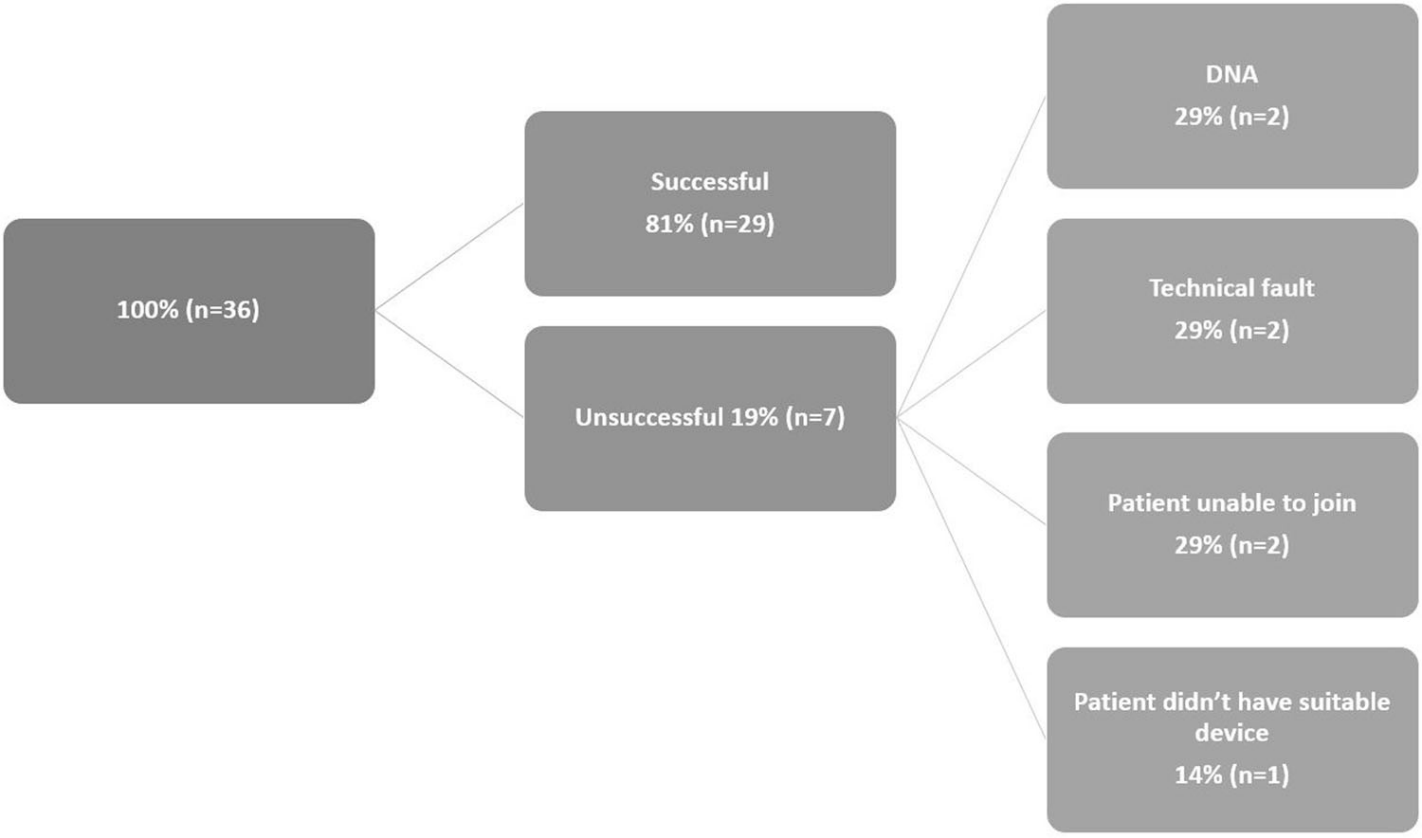
Flowchart of videoconsultations. Reasons for unsuccessful consultations.

VCs were conducted by two senior Ophthalmology trainees. 36 VCs were attempted (January to June 2022). 81% (29/36) of appointments were successful for patients (age range 19–85 years) (Fig. 1).

**Fig. 2:**
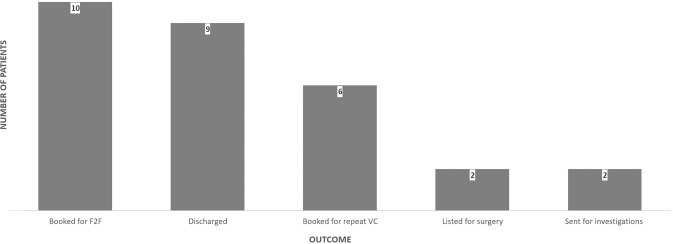
Graph illustrating outcomes of successful videoconsultations.

76% (22/29) of appointments started on time and 86% (25/29) finished punctually. Average duration was 9.7 min (3–29 min). Clinical presentations included lid (76%), orbital (14%) and lacrimal (10%) causes. The majority of patients were booked for F2FC or discharged (Fig. 2).

89% of patients surveyed would recommend VCs to others. 79% preferred VCs to TCs and 54% preferred to F2FC. In 75% of cases, the clinician felt VCs were no less effective than F2FC. In 100% of cases, the clinician and patient found VC “easy” or “very easy” to conduct and join the VC, respectively. Patients preferred the flexibility and personal approach compared to TCs. Of the successful VCs, audiovisual and technical difficulties were experienced in 11%.

We implemented Oculoplastic VCs according to GMC key principles for VCs (https://www.gmc-uk.org/ethical-guidance/learning-materials/remote-prescribing-high-level-principles). The feedback was encouraging. Increasing age did not appear to be a factor in successful Oculoplastic VCs from a study of 187 patients [4]. Our experience reflected this; the ubiquity of devices supporting this platform possibly contributes. Referrals were not triaged prior to assigning VCs. Next, we will conduct VCs for non-emergency new referrals, follow-up for investigation results and after minor procedures. For cases necessitating detailed examination, an asynchronous approach whereby photographs are submitted by patients or community optometrists beforehand could be used [3]. We did not directly compare outcomes with F2FCs, however other peer-reviewed studies show comparable results [3]. Covid-19 introduced a change which is advantageous to service provision. It is cost-effective, achieving patient satisfaction. A next stage would be to develop tailored consensus guidelines with national Oculoplastic societies to ensure safe, effective care.
